# Mucinous Cystic Neoplasms of Pancreas

**DOI:** 10.14740/gr600e

**Published:** 2014-05-02

**Authors:** Shah Naveed, Hasina Qari, Tanveer Banday, Asma Altaf, Mah Para

**Affiliations:** a J&K Health Services, India; b Department of Medicine, Bangalore Medical College, India; c ITS Dental College, Ghaziabad, India; d Govt. Dental College, Srinagar, India

**Keywords:** Pancreatic cystic lesion, Pancreatic mucinous cystic neoplasm, Pancreatic mucin-producing cysts, Pancreatic cystic neoplasm, Pancreatic ovarian-type stroma

## Abstract

The purpose of this study was to investigate the actual management of mucinous cystic neoplasm (MCN) of the pancreas. A systematic review was performed in December 2009 by consulting PubMed MEDLINE for publications and matching the key words “pancreatic mucinous cystic neoplasm”, “pancreatic mucinous cystic tumor”, “pancreatic mucinous cystic mass”, “pancreatic cyst” and “pancreatic cystic neoplasm” to identify English language articles describing the diagnosis and treatment of the MCN of the pancreas. In total, 16,322 references ranging from January 1969 to December 2009 were analyzed and 77 articles were identified. No articles published before 1996 were selected because MCNs were not previously considered to be a completely autonomous disease. Definition, epidemiology, anatomopathological findings, clinical presentation, preoperative evaluation, treatment and prognosis were reviewed. MCNs are pancreatic mucin-producing cysts with a distinctive ovarian-type stroma localized in the body-tail of the gland and occurring in middle-aged females. The majority of MCNs are slow growing and asymptomatic. The prevalence of invasive carcinoma varies between 6% and 55%. Preoperative diagnosis depends on a combination of clinical features, tumor markers, computed tomography (CT), magnetic resonance imaging, endoscopic ultrasound with cyst fluid analysis and positron emission tomography-CT. Surgery is indicated for all MCNs.

## Introduction

True pancreatic cystic lesions account for only 10-15% of all pancreatic cystic lesions and less than 1% of pancreatic tumors [1]. Pancreatic cystic lesions are being increasingly identified with the widespread use of advanced radiological techniques [2]. The incidence of pancreatic cysts (PCs) has been estimated to be between 1% and 2% in patients who had a computed tomography (CT)/magnetic resonance imaging (MRI) performed [3, 4]. Pancreatic cystic neoplasms (PCNs) comprise a different group of histopathologic entities. True pancreatic cystic tumors fall into one of the following types: serous tumors (including serous cystadenoma (SCA) and cystadenocarcinoma), mucinous tumors (including mucinous cystadenomas, mucinous cystadenocarcinomas, intraductal papillary adenomas and intraductal papillary adenocarcinoma) and solid pseudopapillary tumors [1, 5]. In 1978, Compagno et al [6] first classified cystic tumors into serous cystic neoplasms (SCNs) and mucinous cystic neoplasms (MCNs) of the pancreas and identified MCN as a distinct disease occurring almost exclusively in the pancreas body and tail of middle-aged women [6, 7]. Until 1996, when the World Health Organization distinguished between intraductal papillary mucinous neoplasms (IPMNs) and MCNs, emphasizing the presence of ovarian stroma in the latter, and until 1997 when the Armed Forces Institute of Pathology confirmed this distinction, MCN and IPMNs were frequently confused [7-10]. Nowadays, MCN and IPMNs represent two distinct neoplasms with different biologic behaviors, pathologic features and prognosis [11-14].

PCNs are now found with increasing frequency compared to the past due to the improvement and refining of modern imaging techniques like multidetector, three-dimensional CT or MRI, or endoscopic ultrasound (EUS) [15].

The aim of this study was to review the literature to clarify the management of cystic mucinous neoplasm of the pancreas.

## Literature Search

A comprehensive literature review was performed in December 2013 by consulting PubMed MEDLINE for publications, matching the key words of “pancreatic mucinous cystic neoplasm”, “pancreatic mucinous cystic tumor”, “pancreatic mucinous cystic mass”, “pancreatic cyst” and “pancreatic cystic neoplasm” to identify English language articles on MCNs.

Definition, epidemiology, anatomopathological findings, clinical presentation, preoperative evaluation, treatment and prognosis were analyzed.

## Definition and Epidemiology

MCNs are defined as mucin-producing and septated cyst-forming epithelial neoplasia of the pancreas with a distinctive ovarian-type stroma. They are usually solitary and their size ranges between 5 and 35 cm, has a thick fibrotic wall and no communication with the ductal system [14]. MCNs are rare and less common than IPMNs and SCNs in most series [16]. Female to male ratio of 20:1 and a mean age at diagnosis is between 40 and 50 years (range 14 - 95 years) [9, 10, 13, 14]. In 95-98% of cases, the site of the neoplasm is in the body and tail of the pancreas [7, 10, 12, 17, 18]. When the MCNs are localized in the pancreatic head, mucinous cystoadenocarcinoma is more prevalent [10, 13].

Invasive carcinoma incidence in MCN varies between 6% and 36% [11-14, 17, 19].

## Anatomopathological Findings

Macroscopically, MCNs usually appear as solitary, multilocular or unilocular lesions with a mean size of 7 - 8 cm (range 0.5 - 35 cm) with a thick fibrotic wall and containing mucin, even when hemorrhagic, watery or necrotic content is observed [11].

The consensus conference of the International Association of Pancreatology in Sendai (Japan) [11, 12] in 2004 established that the histological presence of unique ovarian-type stroma was mandatory to diagnose MCN as this was not found in other pancreatic neoplasms [13, 16]. MCNs display no communication with the pancreatic ductal system, although some series do suggest that a small proportion of MCNs may show microscopic communication with the pancreatic ducts [20].

Under light microscopy, the cysts are lined by a columnar mucin-producing epithelium and different grades of dysplasia are seen: mild (MCN adenoma), moderate (MCN borderline) and severe (MCN carcinoma *in situ*) [21]. The epithelial lining is positive for CK7, CK8, CK18, CK19, EMA and, less frequently, CK20, DUPAN-2, CEA and CA 19-9 [11, 13]. An invasive adenocarcinoma of the tubular or ductal type is associated in about one-third of cases [9]. The immunophenotype of ovarian-type stroma is similar to the normal ovarian one with positivity for vimentin, calretinin, tyrosine hydroxylase, SMA, α-inhibin, melan-A, CD99 and Bcl-2 and frequently for PR and ER. The origin of ovarian stroma of the pancreas is still being debated [22]. The similarities (namely gender, morphology, stromal luteinization) between pancreatic MCT and its ovarian, hepatobiliary, and retroperitoneal counterparts suggest a common pathway for their development [13]. A stimulation of endodermal immature stroma by female hormones or primary yolk cell implantation in the pancreas has been suggested in literature [13], because buds of the genital tract and dorsal pancreas are adjacent to each other during embryogenesis. Also as the dorsal pancreatic enlargement mainly gives rise to the pancreatic body and tail, this could explain the predilection of MCNs for the distal pancreas [23].

Although the pathologic diagnosis of malignancy is based on invasion of the pancreatic parenchyma or metastases [8], MCNs that do not have conclusive evidence of carcinoma are considered premalignant [10].

A thickened wall with peripheral calcification and papillary proliferations, vascular involvement and hypervascular pattern should be considered as suggestive of MCN with malignant changes [18, 20]. Invasive MCN (mucinous cystadenocarcinoma or MCN with associated invasive carcinoma) is generally a tubular/ductal carcinoma [11], and less common histological variants are represented by undifferentiated carcinoma with osteoclast-like giant cells [24], adenosquamous or colloid cells [25], or sarcomatoid carcinoma [22], carcinosarcoma and choriocarcinoma [11].

The increasing degree of dysplasia and tendency for invasion have been correlated with activating point mutations in the *k-ras* gene and mutations in the *TP5* gene [11].

## Clinical Presentation

The majority of cystic tumors of the pancreas are slow growing and asymptomatic. When symptoms do occur, they are usually secondary to a mass effect and tend to be vague and poorly localized in nature [9, 10]. The majority of MCNs are slow growing and asymptomatic [18]. In a series of 212 consecutive patients with cystic pancreatic lesions, 36.7% were asymptomatic, among whom 17% were SCA, 28% were MCNs, 27% were IPMNs and 2.5% were ductal adenocarcinomas, and 28% had MCNs; in the symptomatic group, 16% had MCNs [26].

The typical clinical appearance is characterized by epigastric heaviness and fullness (60-90%) or by an abdominal mass (30-60%) [10, 13, 17, 26]. Nausea, vomiting (20-30%) and back pain (7-40%) can also be present. These lesions are occasionally discovered in patients scanned for other indications [15, 17, 27].

No specific symptom was significantly associated with a likelihood of malignancy [17], although increasing anorexia and weight loss (10-40%) may be associated with malignant changes [10, 17, 18, 26, 28]. In another study, MCNs were seen mostly in women (95%) and in the distal pancreas (97%); 25% were incidentally discovered. Symptomatic patients typically had mild abdominal pain, but 9% presented with acute pancreatitis. This series, the largest with MCNs defined by ovarian stroma, shows a prevalence of cancer of only 17.5%. Patients having invasive carcinoma were older, possibly suggesting progression from adenoma to carcinoma. One study demonstrated that preoperative clinical characteristic such as patients’ age, male gender, tumor size and presence of symptoms can predict malignancy in the cyst and CT scan is not sufficiently accurate to differentiate among the benign and malignant pancreatic cystic lesions [29].

## Preoperative Evaluation

The main differential diagnosis of MCNs includes other neoplastic cystic lesions (SCN and the IPMNs) and non-neoplastic cystic lesions (pancreatic pseudocysts). There is no single discriminating test, but preoperative diagnosis depends on a combination of modes, including clinical features, tumor markers, CT and MRI, EUS with cyst fluid analysis and positron emission tomography.

High values of CEA and CA 19-9 show a high positive predictive value for pancreatic malignancy or pre-malignancy in the preoperative assessment of pancreatic cystic mass (70-100%) [12, 18, 30]. Positive CEA marker status is an indicator of an MCT, although sensitivity is low at 17%. Using three serum tumor markers (CEA, Ca 19-9 and Ca 125), 27% of MCTs were found to have two or more markers positive, compared to none for the SCTs. Sensitivity decreases to 13% for differentiating benign MCTs from benign SCTs, but specificity remains 100%.

In the differential diagnosis of SCTs vs. MCTs, no reliable serum tumor marker exists which can diagnose SCTs and spare some patients unnecessary operations. Nonetheless, positive CEA serum marker status and/or the presence of more than two positive serum markers (CEA, Ca 19-9, or Ca 125) indicates the presence of an MCT and can prevent delay in diagnosis [30].

A CEA level of more than 400 ng/mL is a good predictor of malignancy in MCNs (sensitivity 45-50%, specificity 95-100% and accuracy 75-80%) [27].

Trans-abdominal ultrasound examination has a low accuracy (50%) for cystic neoplasms of the pancreas [31].

EUS improves that accuracy and allows better evaluation of the wall as it may show separation or nodules within the cyst. Furthermore, EUS can be used to obtain aspiration of the cyst contents and to perform a biopsy of the wall. Cyst fluid amylase concentration of < 250 U/L has been considered capable of excluding pseudocysts of the pancreas (sensitivity 40-45%, specificity 95-100% and accuracy 60-65%), while CEA < 5 ng/mL could suggest a benign etiology (sensitivity 45-50%, specificity 95-100% and accuracy 65-70%) [32]. EUS-FNA cytology and cyst fluid CEA greater than 192 ng/mL show the highest accuracy (79%) for differentiating MCNs from non-MCNs [33, 34]. On the contrary, EUS morphology alone cannot distinguish between the two groups [34].

In any case, the main differential diagnosis of MCNs is with SCNs which have a low CEA in the fluid and an equal distribution throughout the pancreas, with pancreatic pseudocysts (PC) that usually show necrotic debris within the cyst cavity, and with branch duct IPMNs communicating with the ductal pancreatic system and consequently showing elevated cystic fluid amylase [7].

Although pancreatitis may be present in the history of patients with PCNs, when a cyst arises in a patient with chronic pancreatitis, the most frequent diagnosis is PC [28]. On the other hand, when pancreatitis is unexpected and occurs for the first time, the cyst could be a tumor, determining the development of pancreatitis due to compression of the pancreatic duct [35]. This is a crucial problem, because the risk of managing cystic mucinous neoplasms in patients with a prior history of pancreatitis, like pseudocysts by a pseudocyst-jejunal anastomosis or pseudocyst-gastrostomy, is higher than usual, with disastrous long-term prognosis [36]. Diagnostic imaging can help to distinguish MCNs from pseudocysts when there are features of neoplasia; however, no imaging investigation can reliably differentiate the two conditions in all cases. If clinical doubt remains, it is preferable to err on the side of safety and either employ a “wait and watch” strategy or to resect a cystic pancreatic lesion rather than drain a potentially malignant MCN [36].

The demonstration of a solid component, invasion outside the confines of the pancreas, or pancreatic duct obstruction through EUS is highly indicative of malignancy with sensitivity, specificity and accuracy of 70%, 100% and 60%, respectively [27]. However, in the absence of these findings, the ability of EUS to diagnose malignancy is limited with an overall sensitivity, specificity and accuracy of 56%, 45% and 51%, respectively [34]. The added advantage of EUS in performing aspiration of cyst content and sampling of the cyst wall and septa or mural nodules is that it allows small lesions as well as suspicious areas to be analyzed. Laparoscopic and intraoperative ultrasounds are highly operator dependent with an accuracy ranging from 40% to 90% [7].

Multidetector CT and magnetic resonance cholangiopancreaticography (MRCP) play a critical role in assessment, defining size, septation, calcifications, nodules of the wall and communication with the ductal system of the PC.

At cross-sectional imaging, the MCN appears as a unilocular or multilocular single macrocyst with a solid component, with no communication with the main duct [27].

Recently, Kim et al [37] defined some significant CT features for differentiating MCNs from SCNs and IPMNs: the shape is smooth in MCNs, multicystic and lobulated in SCNs, and pleomorphic and clubbed finger-like in IPMNs; the main pancreatic duct is not dilated or proximally only in SCNs, and if dilated, whole in IPMN.

In spite of the improvement in pancreatic tumor visualization resulting from CT and MRI, the ability to perform diagnosis of these techniques individually, as well as EUS, remains poor (25-30%) [9]. In a multivariate analysis by Visser et al [19] in 2008, the combination of CT and MRI data showed an accuracy ranging from 44% to 83%.

Cross-sectional imaging generally shows peripheral calcification, a thickened wall, papillary proliferations, vascular involvement and hypervascular pattern in the cases of malignant MCNs [38]. Although peripheral eggshell calcification is not easily detected by CT, this is a specific feature of the MCNs and is highly predictive of malignancy [38].

The clinical value of MRCP is similar to endoscopic retrograde cholangiopancreatography or percutaneous transhepatic cholangiography [39].

In spite of a complete diagnostic assessment, the surgeon’s preoperative diagnosis is correct in one-third of cases, incorrect in another third and non-specific in the remainder [9, 19].

## Treatment

Surgical excision is indicated for all MCNs considered pre-malignant. Factors influencing treatment include tumor histological features, the patient’s age and surgical risk, and tumor size and location.

### Left pancreatectomy

Because mucinous cystic adenomas of the pancreas are usually localized at the level of the body and tail of the pancreas, the most common operation performed to cure these neoplasms is distal pancreatectomy which is a safe procedure in high volume centers (overall postoperative morbidity ranging from 5% to 50% and a mortality rate of 0%) [7, 10, 17]. The main complication, pancreatic fistula, occurs in 15-20% of cases [40].

In patients with MCNs of < 4 cm without mural nodules, parenchyma sparing resections (middle pancreatectomy) and distal pancreatectomy with spleen preservation as well as laparoscopic procedures should be considered [41].

MCNs affecting the pancreatic neck or the proximal body could be managed either by an extended right or, more frequently, by an extended left pancreatectomy. These extended resections of normal pancreatic tissue may induce endocrine and exocrine insufficiency respectively in 30-35% and 15-20%, which in a benign or premalignant disease could be discussable [32].

### Middle pancreatectomy

Middle pancreatectomy can be considered in the surgical management of MCNs located at the level of the pancreatic proximal body or neck, preserving endocrine and exocrine function with respect to extended left pancreatectomy or pancreaticoduodenectomy, and also preserving the spleen.

The main pitfalls of this technique are the technical difficulty, the higher incidence of postoperative complications and the risk of recurrence from potentially residual neoplasm [12].

### Enucleation

Because the probability of malignancy in patients with MCNs smaller than 2 cm without nodules is very low, enucleation could be performed to avoid postoperative pancreatic insufficiency [17]. This procedure is proposed for patients with MCNs smaller than 2 cm with benign features and superficially located [32]. Enucleation can be performed without risk of recurrence but has been associated with a higher incidence of pancreatic fistula (30-50%) [42].

### Whipple procedure

A major oncologic resection, applying a Kausch-Whipple or pylorus-preserving technique, is recommended for MCNs that are localized monocentrically in the head.

### Lymphadenectomy

Pancreatectomy with lymph node dissection is necessary when an invasive carcinoma is suspected. Although the preoperative and intraoperative assessment of the grade of invasiveness is often difficult, whenever any doubt exists typical resection with lymph node dissection must be pursued [12]. There is no evidence in literature of invasive mucinous cystic adenocarcinoma with distant lymph node metastases, so only a loco-regional lymphadenectomy is justified [7, 17]. Because the probability of malignancy is very low in the cases of small MCNs without nodules, lymphadenectomy can be avoided [7].

### Laparoscopy

In the cases of benign-appearing and small malignant lesions (< 5 cm), a minimally invasive approach may be considered [17]. Recent experiences from high-volume centers demonstrate that the laparoscopic approach for distal pancreatectomy for MCNs of the body and tail of the pancreas is feasible and safe [43].

### Chemotherapy

Gemcitabine (GEM) is the standard therapy for advanced pancreatic cancer [44]. Its effectiveness against advanced MCNs has been reported [45, 46].

Recently, some combinations have been reported to be superior to GEM alone [45]. GEM-oxaliplatin treatment has been proposed to be more effective in terms of clinical progression-free survival [45].

Other modest but interesting advances have been provided by combinations such as GEM-capecitabine and GEM plus a platinum salt [47]. In spite of this, survival results remain disappointing.

### Conservative treatment

A conservative management with regular follow-up has been proposed in the presence of asymptomatic cystic lesions of the pancreas smaller than 3 cm without mural nodules, because the reported risk of malignancy in these cysts was found to be 3% [14, 17, 48]. The suggested follow-up consisting of cross-sectional imaging and FNA cytology should be performed every 6 months for a period of 2 years and yearly after that. This should be continued for at least 4 years and then the interval of follow-up can be lengthened after 6 years of no change [48]. When the cyst enlarges or when symptoms occur (in up to 20% of patients after follow-up), surgery is mandatory. The reported incidence of the subsequent resection due to change of the clinical, radiological and biochemical features of the lesions after initial conservative treatment was 4-10% and malignancy rate in these cases was 3% [49].

## Prognosis

After resection, in the absence of invasive carcinoma, prognosis of MCNs is excellent, with an overall survival rate of 100% [8-10, 13, 17] and patients do not need follow-up, since several studies have shown that the risk of recurrence following resection is 0% [14]. Patients with invasive mucinous cystadenocarcinoma, show a 5-year survival rate of 20-60%, which is much better than that for non-MCN-associated ductal adenocarcinoma [8-11, 14, 16, 17, 23].

## Conclusion

Although the histological distinction between MCNs and IPMNs, through the identification of ovarian stroma initially, is very important in clinical practice, the management of MCNs has not yet been standardized and continues to evolve.

The approach to patients with suspected MCN is based on EUS and cross-sectional imaging in association with FNA cytology, detecting an incidence of correct differentiation between MCNs and non- MCNs of 75%.

Because at present we are unable to identify the benign MCNs that will progress into invasive carcinoma, all MCNs should be resected, regardless of size, in patients who are fit candidates for surgery, because surgery is routinely curative in the cases of non-invasive tumor. Moreover, the non-operative management based on periodic CT or MRI requires years of careful follow-up with a high cost of imaging and the enucleation technique carries the risk of non-oncological radicality. In patients with non-invasive MCN after complete anatomic resection, postoperative surveillance is unnecessary.
